# Identification of Genes Associated with Familial Focal Segmental Glomerulosclerosis Through Transcriptomics and In Silico Analysis, Including *RPL27*, *TUBB6*, and *PFDN5*

**DOI:** 10.3390/ijms252111659

**Published:** 2024-10-30

**Authors:** Anfal Hussain Mahmoud, Reem Sami Alhamidi, Burcu Yener Ilce, Alaa Mohamed Hamad, Nival Ali, Amjad Mahasneh, Iman M. Talaat, Abdelaziz Tlili, Rifat Hamoudi

**Affiliations:** 1 Research Institute for Medical and Health Sciences, University of Sharjah, Sharjah P.O. Box 27272, United Arab Emirates; u20104731@sharjah.ac.ae (A.H.M.); ralhamidi@sharjah.ac.ae (R.S.A.); bilce@sharjah.ac.ae (B.Y.I.); alaahamad607@gmail.com (A.M.H.); nali@sharjah.ac.ae (N.A.); or r.hamoudi@ucl.ac.uk (R.H.); 2 Department of Clinical Sciences, College of Medicine, University of Sharjah, Sharjah P.O. Box 27272, United Arab Emirates; 3 Department of Biology, Chemistry and Environmental Sciences, American University of Sharjah, Sharjah P.O. Box 26666, United Arab Emirates; amahasneh@aus.edu; 4 Department of Biotechnology and Genetic Engineering, Jordan University of Science and Technology, Irbid 22110, Jordan; 5 Pathology Department, Faculty of Medicine, Alexandria University, Alexandria 21131, Egypt; 6 Department of Applied Biology, College of Sciences, University of Sharjah, Sharjah P.O. Box 27272, United Arab Emirates; 7 Center of Excellence for Precision Medicine, Research Institute of Medical and Health Sciences, University of Sharjah, Sharjah P.O. Box 27272, United Arab Emirates; 8 BIMAI-Lab, Biomedically Informed Artificial Intelligence Laboratory, University of Sharjah, Sharjah P.O. Box 27272, United Arab Emirates; 9 ASPIRE Precision Medicine Research Institute Abu Dhabi, University of Sharjah, Sharjah P.O. Box 27272, United Arab Emirates; 10 Division of Surgery and Interventional Science, University College London, London NW3 2QG, UK

**Keywords:** Focal segmental glomerulosclerosis (FSGS), Minimal change disease (MCD), genetic, glomerular, tubulointerstitial

## Abstract

Focal segmental glomerulosclerosis (FSGS) is a major cause of nephrotic syndrome and often leads to progressive kidney failure. Its varying clinical presentation suggests potential genetic diversity, requiring further molecular investigation. This study aims to elucidate some of the genetic and molecular mechanisms underlying FSGS. The study focuses on the use of bioinformatic analysis of gene expression data to identify genes associated with familial FSGS. A comprehensive in silico analysis was performed using the GSE99340 data set from Gene Expression Omnibus (GEO) comparing gene expression in glomerular and tubulointerstitial tissues from FSGS patients (*n* = 10) and Minimal Change Disease (MCD) patients (*n* = 8). These findings were validated using transcriptomics data obtained using RNA sequencing from FSGS (*n* = 3) and control samples (*n* = 3) from the UAE. Further validation was conducted using qRT-PCR on an independent FFPE cohort (FSGS, *n* = 6; MCD, *n* = 7) and saliva samples (FSGS, *n* = 3; Control, *n* = 7) from the UAE. Three genes (*TUBB6*, *RPL27*, and *PFDN5*) showed significant differential expression (*p* < 0.01) when comparing FSGS and MCD with healthy controls. These genes are associated with cell junction organization and synaptic pathways of the neuron, supporting the link between FSGS and the neural system. These genes can potentially be useful as diagnostic biomarkers for FSGS and to develop new treatment options.

## 1. Introduction

Focal segmental glomerulosclerosis (FSGS) is a significant cause of nephrotic syndrome, responsible for 40% of cases in adults and 20% in pediatric patients [1]. Moreover, this disease is the leading cause of end-stage renal disease in the United States [2], and is the most common type of glomerulonephritis, followed by membranous nephropathy [3]. FSGS is among the most prevalent causes of primary glomerulopathy in adults [2]. Despite limited data, it is evident that FSGS significantly contributes to the overall burden of kidney disease and is considered a significant global health concern that is often underestimated. FSGS requires improved diagnosis, increased treatment accessibility, and ongoing research [4].

**Figure 1:**
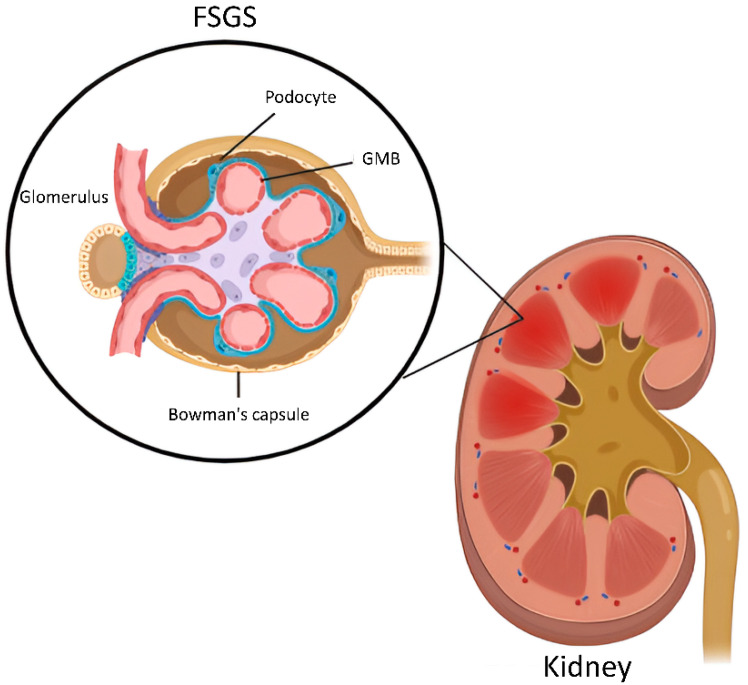
Progression of focal segmented glomerulosclerosis. The image shows the development of focal segmental glomerulosclerosis (FSGS), which initiates with the detachment of specialized glomerular visceral epithelial cells called podocytes from the glomerular basement membrane (GBM). This causes the GBM to protrude outward and adhere to the inner lining of Bowman’s capsule. The inflamed region affected by FSGS is highlighted in dark red. The surrounding healthy kidney tissue is shown in light red. This illustration was generated by biorender.com.

FSGS occurs when podocytes, specialized glomerular visceral epithelial cells, detach from the glomerular basement membrane (GBM), as shown in Figure 1. The reduction in podocyte numbers attached to the GBM results in decreased hydrostatic pressure between the interglomerular capillaries and podocytes, resulting in the GBM protruding outward and attaching to the inner lining of Bowman’s capsule [5]. Consequently, parts of the glomerulus, a bundle of tiny blood capillaries encapsulated in the Bowman Capsule that serves as a kidney filter, will develop scarring (sclerosis) [5,6]. Furthermore, this podocyte defect will result in protein leakage from the blood into the urine, leading to kidney failure [7]. FSGS is known for its diverse presentation, characterized by distinct histological patterns. This variability can be attributed to specific genetic variants affecting different cellular pathways regulating the functions of various kidney parts [8]. 

FSGS is classified as primary (which occurs suddenly because of a circulatory factor in the blood), secondary (caused by HIV infection, maladaptive response or is drug-induced), or familial FSGS (caused by a mutation in one or more vital genes). Although these subclasses share some clinical and histologic characteristics, their prognosis and management differ considerably [5].

Familial FSGS can arise from genetic causes that manifest as sporadic or familial diseases [6]. It may be inherited in an autosomal dominant, autosomal recessive, X-linked, or mitochondrial manner [9]. The inherited mutations affect the structure and functions of crucial podocyte proteins. Some examples of these podocyte-related genes are *NPHS1*, *NPHS2*, *CD2AP*, *PLCE1* and *TRPC6* [10]. Other genes associated with FSGS affect the regulation of the actin cytoskeleton, which plays a role in maintaining podocyte structure and function. These genes include *ACTN4*, *MYO1E*, *ANLN*, *ARHGDIA*, *ARHGAP24*, *INF2* and *KANK2* [8]. Nuclear pore complex genes, including *NUP93*, *NUP205*, *XPO5*, *NUP107*, and *PAX2*, have also been implicated in FSGS [8]. Additionally, mutations in genes encoding membrane-associated proteins such as *PTPRO*, *EMP2*, *PODXL*, and *LAMA5* can cause FSGS [2,8].

Familial FSGS presents in early childhood; nevertheless, as additional mutations associated with FSGS are identified, the significance of genetic FSGS onset in adulthood is increasing. Furthermore, family members who share the same mutation may exhibit varying phenotypes, suggesting that environmental, genetic, or epigenetic factors play a crucial role [5]. FSGS patients with unidentified causes are classified as having “unknown” forms of the disease. Investigating familial occurrences of the disease could provide valuable information regarding the genetic factors underlying the disease in FSGS patients with unknown causes [5].

FSGS is diagnosed by integrating clinical history, laboratory data, and renal histopathology [2]. Distinguishing between various types of FSGS can be aided by identifying the features of podocyte foot process effacement (FPE) using electron microscopy. Because this method may not reliably detect cases of genetic FSGS, genetic analysis, including next-generation DNA sequencing, is utilized to screen multiple genes simultaneously in a cost-effective manner [11]. However, determining the pathogenicity of a detected genetic variant can be challenging [12].

FSGS presents a significant challenge for diagnosis and treatment due to the absence of definitive biomarkers. The disease is often complex to detect early during childhood, especially in the case of familial FSGS, and it is challenging to distinguish it from other related diseases, such as minimal change disease (MCD) [13]. FSGS and MCD are two primary glomerular diseases associated with acute-onset nephrotic syndrome. FSGS is distinguished by segmental sclerotic lesions under light microscopy, in contrast to MCD. These lesions in FSGS are usually localized in the corticomedullary region of the kidney. Procuring sufficient kidney tissue for biopsy can be challenging, thus limiting the ability to establish a definitive diagnosis. These diseases demonstrate varying prognoses and responses to treatment, underscoring the significance of accurate differentiation for appropriate management [14]. Transcriptomic studies have shown promise in improving the detection of biomarkers to enhance disease treatment [15].

Therefore, in this study, we aimed to identify key genes linked to FSGS and MCD. For this, we utilized a specialized in-house bioinformatics pipeline to identify unique transcriptomics profiles and differentially expressed genes in the glomerular and tubulointerstitial tissues affected by FSGS progression and compared with MCD using publicly available transcriptomics data from German patients. Furthermore, these findings were validated on whole transcriptomics data using RNA sequencing obtained from the saliva of FSGS patients and healthy controls in the UAE. The identified potential biomarkers were validated using qRT-PCR analysis using RNA obtained from formalin-fixed paraffin-embedded (FFPE) samples from kidney biopsies and the saliva of FSGS and MCD patients in the UAE and compared to healthy controls. In addition, to investigate the putative pathways related to FSGS, we screened an affected family from the UAE using whole exome sequencing (WES).

This approach aims to provide novel insights into the molecular mechanisms underlying FSGS progression, facilitating the identification of crucial diagnostic, prognostic and therapeutic targets to improve FSGS patient management.

## 2. Result

### 2.1. In Silico Data Normalization and Filtration for FSGS Data Set

**Figure 2:**
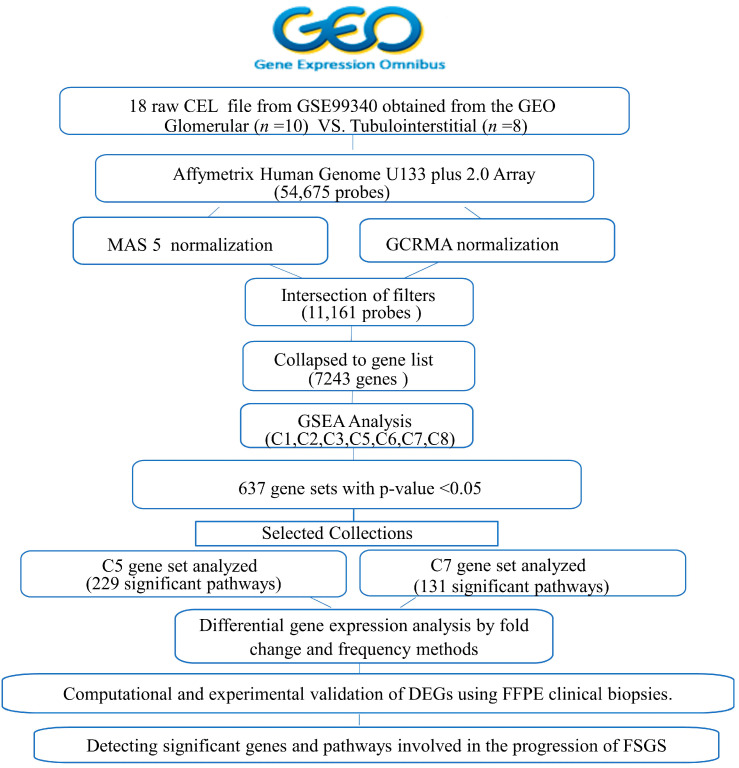
Flow chart of in silico data normalization and filtration.

The process of normalizing and filtering transcriptomic data for FSGS is illustrated in Figure 2. The study involved a glomerular tissue analysis of 10 FSGS patients compared to the tubulointerstitial tissue from three FSGS and five MCD patients. Subsequently, after applying the MAS5 and GCRMA filters, 11,161 probes were retained for further analysis. As stated in the method section, these filtered probes aligned with the 7243 genes in GSEA. First, the process started by obtaining 18 CEL files from the GSE99340 data set in the GEO database, comprising ten samples for glomerulus and eight for tubulointerstitial tissue. Subsequently, GSEA was conducted to identify the key genes and upregulated pathways in FSGS and MCD patients across the two tissue types.

### 2.2. Gene Set Enrichment Analysis Revealed Changes in the Activated Cellular Pathways Related to Ontology and Immunological Signature Genes in FSGS Patients

The study involved two groups of patients, namely, FSGS and MCD, and utilized absolute gene set enrichment analysis (absGSEA) to process the samples. The aim was to identify differentially activated pathways between the glomerular and tubulointerstitial tissue of the two groups. Differentially activated pathways were obtained using cutoff criteria of *p*-value < 0.05 and false discovery rate (FDR) < 0.25.

**Figure 3:**
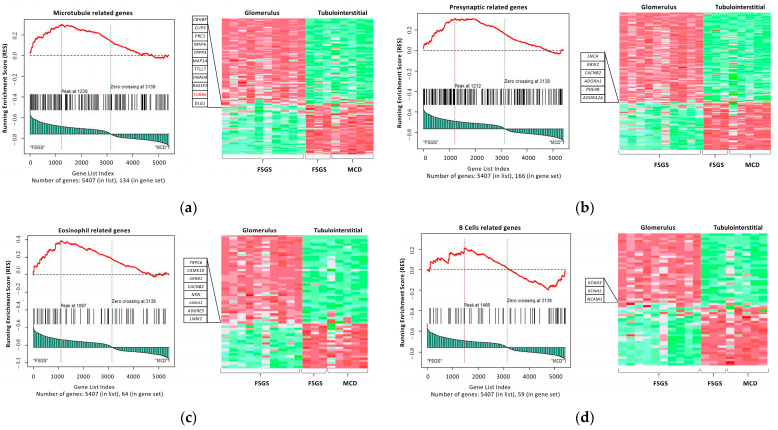
In silico data for GSEA-enriched pathways. Illustrates the GSEA (**a**,**b**) output for datasets C5: ontology and (**c**,**d**) C7,: immunologic signature gene sets, comparing FSGS and MCD. In this comparison, FSGS is characterized by its focus on the glomerulus, whereas MCD focuses on the tubulointerstitial, with some inclusion of FSGS patients. The similarity of the tubulointerstitial characteristics in both groups led us to utilize this tissue from both FSGS and MCD for comparison with the glomerular tissue of FSGS. The heat map highlights higher expressions in red, while lower expressions are indicated in green.

The in silico analysis identified 229 differentially activated pathways from the biological process, cellular component, and molecular function ontology gene set and 131 activated pathways from the immunological signature gene set. Table 1 lists the number of significant pathways enriched in glomerular tissue compared to tubulointerstitial tissue from absolute GSEA analysis. Some of the most significantly enriched pathways are those involved in glomerulonephritis, muscle contraction, regulation of lymphocyte differentiation, presynaptic membranes, cell junction organization, heart processes, and cardiac chamber development. The complete set of enriched pathways is listed in Supplementary Table S1. Additionally, an example representation of each dataset’s output from the gene set analysis is shown in Figure 3.

### 2.3. RNA Sequencing Identified Alterations in the Activated Cellular Pathways Associated with Ontology and Immunological Signature Genes in FSGS Patients

**Figure 4:**
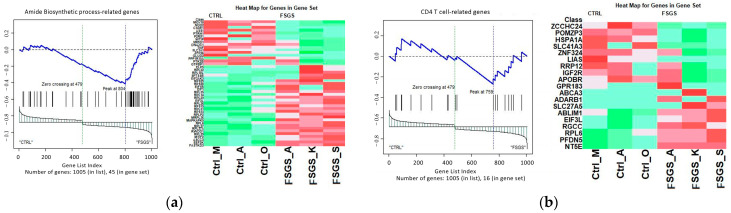
Absolute GSEA-enriched pathways. An example of each dataset’s output from the gene set analysis. (**a**) C5: Ontology and (**b**) C7: Immunologic signature gene sets, respectively. The heat map highlights higher expressions in red, while lower expressions are indicated in green.

Absolute GSEA using the RNAseq data revealed around eleven differentially activated pathways from the biological process, cellular component, and molecular function ontology gene set and five activated pathways from the immunological signature gene set. The most significantly enriched pathways included the amide biosynthetic process, organonitrogen compound biosynthetic process, regulation of cellular stress response, and presynaptic pathway. The complete set of enriched pathways is available in Supplementary Table S2. Additionally, Figure 4 provides an example representation of each dataset’s output from the gene set analysis.

### 2.4. GSEA Identified Differentially Expressed Genes (DEGs) in FSGS Compared to MCD Patients and Healthy Controls

The enriched pathways identified through GSEA underwent additional statistical analysis. Genes that demonstrated a high frequency across all activated cellular pathways (with a *p*-value < 0.05) were chosen. The top 40 genes were selected from in silico analysis (Table 2) and 20 genes were selected from RNA sequencing (Table 3).

### 2.5. Common Genes and Pathways Between In Silico and RNA Sequencing

**Figure 5:**
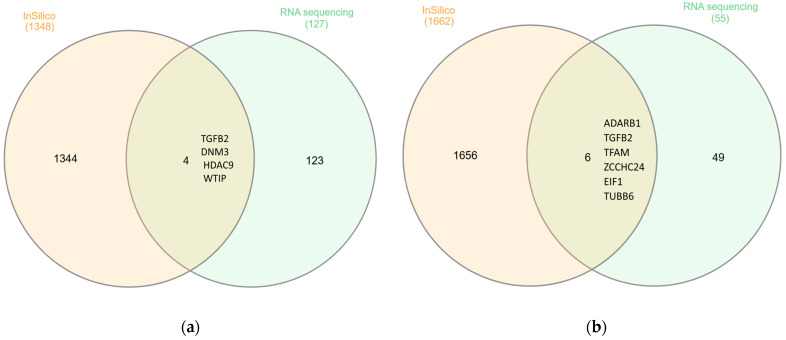
Comparison between in silico and RNA sequencing analyses of FSGS using InteractiVenn. (**a**) In the C5 dataset, four genes (*TGFB2*, *DNM3*, *HDAC9*, *WTIP*) were common to both. (**b**) In the C7 dataset 6 genes (*ADARB1*, *TGFB2*, *TFAM*, *ZCCHC24*, *EIF1*, *TUBB6*) were common to both “http://www.interactivenn.net (accessed on 29 June 2024)”.

InteractiVenn “http://www.interactivenn.net (accessed on 29 June 2024)” was used to compare the genes obtained from the in silico and RNAseq data, and approximately 10 genes were commonly upregulated in both datasets. In the C5 dataset, four genes (*TGFB2*, *DNM3*, *HDAC9*, *WTIP*) were common in the in silico and RNAseq (Figure 5a). In the C7 dataset, six genes (*ADARB1*, *TGFB2*, *TFAM*, *ZCCHC24*, *EIF1*, *TUBB6*) were common in both in silico and RNAseq (Figure 5b).

### 2.6. TUBB6, PFDN5, and RPL27 Genes Showed the Most Significant Fold Change in Expression Between FSGS and Control

**Figure 6:**
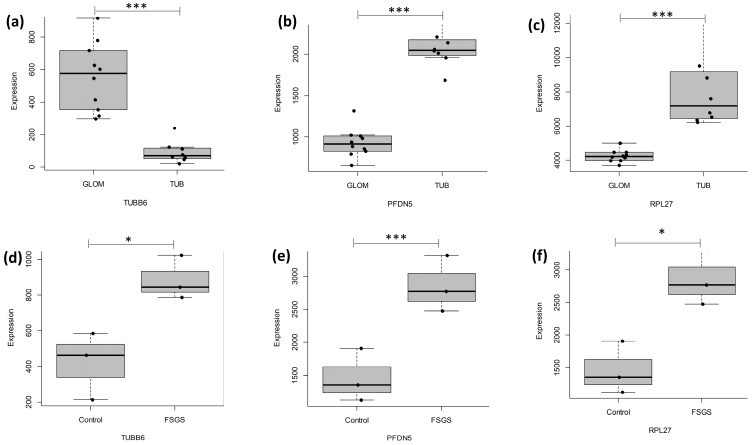
Comparative gene expression analysis of FSGS and MCD. The figure compares the expression levels of three genes (*RPL27*, *TUBB6*, and *PFDN5*) in FSGS and MCD tissues. The in silico study examined gene expressions in glomerular and tubulointerstitial regions, revealing that (**a**) *TUBB6* had higher expression in FSGS (glomerular), whereas (**b**) *PFDN5* and (**c**) *RPL27* had lower expression in FSGS (glomerular). The RNA sequencing analysis (**d**–**f**), comparing controls to FSGS, demonstrated higher expression levels of all three genes in FSGS patients. GLOM; glomerulus, TUB; tubulointerstitial, FSGS: Focal segmented glomerulosclerosis. *p* value. * *p* < 0.05, *** *p* < 0.01.

The fold change in expression of highly expressed genes (*TUBB6*, *PFDN5*, *RPL27*) was derived from the in silico microarray data and graphed to analyze the differential expression pattern between ten samples of focal segmental glomerulosclerosis (FSGS) patients compared to five samples of MCD patients (Figure 6a–c), as well as from the RNAseq data of three FSGS patient samples compared to three healthy control samples, (Figure 6d–f). The study identified a shared pathway—the presynaptic pathway related to the neural system—between the in silico analyses and RNAseq data.

### 2.7. RPL27, TUBB6, and PFDN5 Were Associated with FSGS in the In Vivo Validation Using FFPE Samples from FSGS Patients Compared to MCD Patients

**Figure 7:**
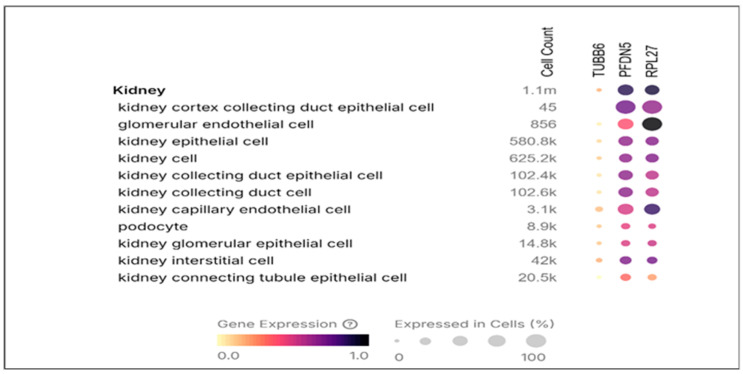
*TUBB6*, *PFDN5*, and *RPL27* gene expression levels in different kidney-specific cells. These data were obtained from primary data submitted to CZ CELLxGENE Discover. Source: “https://cellxgene.cziscience.com/gene-expression (accessed on 13 October 2024)”.

According to CZ CELLxGENE, *RPL27*, *TUBB6*, and *PFDN5* are expressed in various kidney-specific cells, including podocytes (Figure 7).

**Figure 8:**
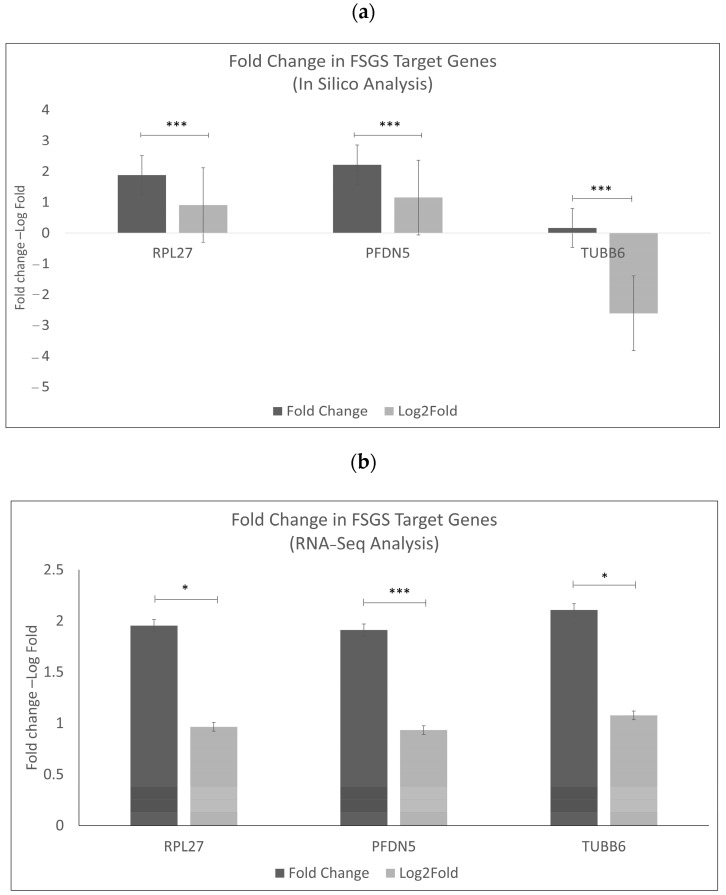
Illustrations of the fold changes in *RPL27*, *TUBB6*, and *PFDN5* were obtained from RNA sequencing analysis and in silico. (**a**) Presentation of the corresponding fold change in FSGS target genes from in silico analysis and (**b**) RNAseq data. *p* value. * *p* < 0.05, *** *p* < 0.01.

**Figure 9:**
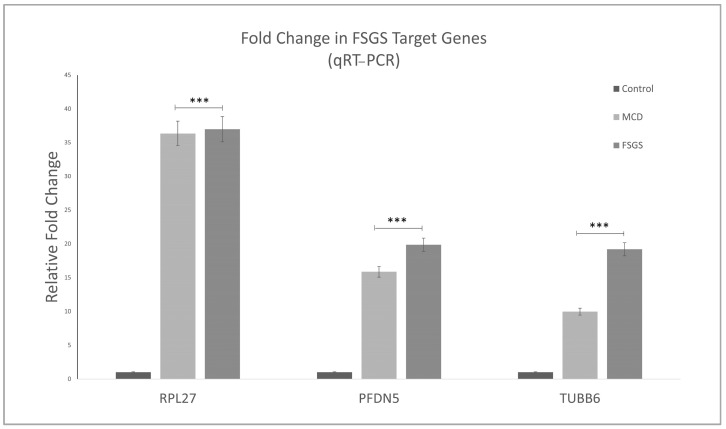
Relative fold changes in three significant genes, *TUBB6*, *RPL27*, and *PFDN5*, were obtained from qPCR. The results demonstrate that FSGS and MCD patients show elevated expression of these three genes compared to healthy controls. *p* value. *** *p* < 0.01.

To further validate our results, the expression values of these three genes identified from in silico and RNAseq analyses (Figure 8) were examined using FFPE samples from six FSGS and seven MCD patients compared to seven healthy controls. Following qPCR, the results indicated that the genes *RPL27* (*p*-value < 0.01), *TUBB6* (*p*-value < 0.05, *p*-value < 0.01), and *PFDN5* (*p*-value < 0.01) exhibited a high fold change in FSGS and MCD compared to healthy controls, confirming their association with FSGS, as shown in Figure 9.

### 2.8. Whole Exome Sequencing Detected Novel Mutations Associated with the Glomerulus

Whole exome sequencing (WES) conducted in the affected family revealed 82 rare variants that potentially segregate with the disease. These variants were heterozygous in all affected individuals and absent in the father’s genome. Among these, one variant was located in the 5′ UTR region, two were in-frame variants, and 35 were missense variants, see Supplementary Table S3.

**Figure 10:**
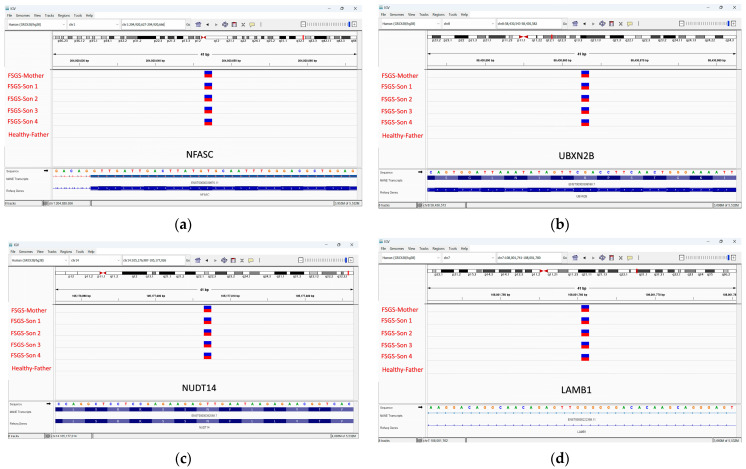
WES identified novel mutations in FSGS patients. In a family with FSGS, all patients were found to be heterozygous for (**a**) a significant mutation in the 5′ UTR of the *NFASC* gene (c.-184T>C). (**b**) Additionally, a novel missense mutation was identified in the *UBXN2B* gene (c.233G>A) and (**c**) the *NUDT14* gene (c.146A>T), along with (**d**) an intronic mutation in the *LAMB1* gene (c.38-28C>A). These mutations were significant in the GSEA analysis.The figure illustrates an Integrated Genomics Viewer (IGV) representation of a heterozygous mutation. The blue color represents the wild-type sequence, whereas the red color indicates the mutant sequence.

In addition, 18 novel mutations (Table 4) showed significance in the in silico study. In the GSEA results, four mutations showed high significance: a variant in the 5′ UTR region *NFASC* (c.-184T>C), missense mutations in *UBXN2B* (c.233G>A) and *NUDT14* (c.146A>T), and an intronic mutation in *LAMB1* (c.38-28C>A), Figure 10. The analysis revealed that the affected mother and her four sons carried these variants, suggesting an autosomal dominant inheritance pattern and providing insight into the genetic basis of FSGS in this family.

## 3. Discussion

In this research, a comprehensive method was employed to examine the genetic causes of FSGS and the underlying molecular mechanisms of the disease. The criteria for selecting target genes included identifying genes with increased expression levels based on data from in silico and RNAseq analyses. Genes with clear and interpretable expression patterns were included, suggesting their potential involvement in the biological processes associated with FSGS progression. In addition to integrating the top-ranking genes identified through (GSEA) from the C5 and C7 gene sets, among others, such as C1, C2, C3, C6, and C8 gene sets. The C5 gene set focuses on biological processes, whereas the C7 gene set consists of immunological pathways. The genes identified were most prominent in the C7 collection, indicating their potential relevance to immunological pathways related to innate and adaptive immunity and kidney function. This comprehensive approach aimed to select target genes with increased expression and significant associations with known biological processes and pathways, providing a robust basis for further investigation.

The selected genes were further validated using FFPE samples from FSGS and MCD compared to healthy controls. Three genes (*TUBB6*, *RPL27*, and *PFDN5)* emerged as significant genes related to FSGS and MCD. The *TUBB6* gene was a substantial biomarker distinguishing between the FSGS and MCD patients in the in silico dataset from European ancestry. However, the *RPL27* and *PDFN5* genes were highly substantial biomarkers in the RNAseq data from the UAE population. This suggests that *TUBB6* may be used as a potential biomarker to compare two related kidney diseases (FSGS and MCD), and *TUBB6*, *RPL27*, and *PFDN5* can be used as potential biomarkers to detect FSGS. These novel genes have not been previously associated with FSGS. This discovery could significantly impact our understanding of the genetic basis of FSGS and may lead to new diagnostic and therapeutic strategies.

Tubulin, specifically the TUBB6 protein, plays a crucial role in the structure of microtubules, cylindrical formations made up of linear protofilaments consisting of alpha- and beta-tubulin heterodimers. Microtubules are involved in critical cellular functions such as mitosis, intracellular transport, the shaping of neurons, and the movement of cilia and flagella [16]. Previous studies have indicated that *TUBB6* may play a significant role in brain development, especially in guiding and extending axons. This is supported by the understanding that other types of tubulin affect these processes and that the expression pattern of *TUBB6* corresponds to early neuronal development. Furthermore, genetic variations associated with *TUBB6* have been linked to decreased stability in microtubules, providing additional evidence for its involvement in brain development [17]. Significantly, their discoveries align with research findings that demonstrate a connection between neural pathways and FSGS through these individuals’ heightened activity in the presynaptic pathway [18].

The *RPL27* gene, which encodes the ribosomal protein L27, was significantly upregulated in the RNAseq analysis and the FFPE samples of FSGS and MCD patients. Ribosomes are essential organelles with diverse functions, primarily translating mRNA into proteins and regulating cellular metabolism. Ribosomal proteins (RPs), the building blocks of ribosomes, serve various functions across different species. They play crucial roles in ribosome assembly and function and serve as scaffolds to enhance the catalytic capability of ribosomal RNA in protein synthesis. Some ribosomal proteins also have additional functions related to DNA repair, transcriptional regulation, and apoptosis. Previous research has shown that ribosome biogenesis involves housekeeping functions and is crucial in the cell cycle and various essential cellular processes [19].

In addition, specific ribosomal proteins, including *RPL27* and *RPS6*, have been identified as highly connected genes, known as hub genes. *RPS6* was upregulated in a separate group of FSGS patients, and increased rpS6 phosphorylation was observed in podocytes of FSGS patients and a mouse model with induced FSGS [20]. Furthermore, previous studies have shown that inhibiting *RPS6* phosphorylation can alleviate podocyte hypertrophy and hinder the progression of FSGS [20]. Despite *RPS6* not being our target gene, its classification as a hub gene, upregulation in FSGS patients, association with podocyte dysfunction, and positive therapeutic impact on podocyte hypertrophy indicate that ribosomal genes warrant further investigation and may hold promise as a therapeutic target for FSGS.

*PFDN5*, also known as prefoldin-5, is a gene that was found to be upregulated in RNAseq analysis. This gene encodes a subunit of the prefoldin protein, a heterohexameric chaperone protein. This protein binds explicitly to cytosolic chaperonin and facilitates the transfer of target proteins [21]. Research by Vainberg et al. (1998) demonstrated that deletion of *PFDN5* in yeast led to impaired functions of the actin and tubulin-based cytoskeleton and disrupted microtubule organization. Additionally, *PFDN5* has been associated with the Wnt/beta-catenin signaling pathway via Wnt4. High *PFDN5* levels are correlated with elevated Wnt4 and increased Wnt/beta-catenin signaling, which have been implicated in the dysregulation of homeostasis observed in FSGS [22].

The Wnt/β-catenin signaling pathway is crucial in causing oxidative stress-induced podocyte injury and proteinuria. Targeting this pathway could be a potential therapeutic approach for proteinuric kidney diseases such as FSGS [23]. Additionally, this pathway promotes renal fibrosis, suggesting that controlling it could be a potential therapeutic target for kidney diseases [24].

The in silico study comparing FSGS and MCD revealed changes in pathways related to glomerulonephritis, muscle function, the regulation of lymphocyte development, the organization of cell junctions, heart development, chamber formation, and immunological pathways. Conversely, RNA sequencing analysis revealed significant changes in several cellular pathways between FSGS patients and healthy controls. These variances encompassed processes such as amide biosynthesis, organonitrogen compound biosynthesis, the regulation of cellular stress response, and genes associated with immune function.

Interestingly, the in silico and RNA sequencing analysis identified a shared disrupted pathway—the presynaptic pathway—suggesting a potential correlation between neuronal signaling and renal function. This pathway is crucial in synaptic transmission, neuromodulation, and neuroendocrine signaling [25]. Although the exact mechanisms of the presynaptic pathway in FSGS are not fully understood, potential connections include neurogenic inflammation, dysfunction of the autonomic nervous system, and the regulation of neurotrophic factors [26]. Recent research has shown a correlation between FSGS and neurodegenerative disorders, which aligns with our findings [27].

The presynaptic pathway is a crucial component of neuronal communication, involving the transmission of chemical signals (neurotransmitters) between neurons at synapses [28]. The neurotransmitter dopamine is essential for regulating blood pressure and kidney function. Imbalances in dopamine levels can lead to salt-sensitive hypertension and an imbalanced renin-angiotensin system [29]. Serotonin, found in the central nervous system (CNS) and the kidneys, regulates vascular resistance, glomerular functions, and mitochondrial homeostasis [30]. Serotonin dysregulation has been linked to neurological disorders of the CNS [28]. The CNS also influences water reabsorption by controlling vasopressin release from the hypothalamus/posterior pituitary gland. Norepinephrine released from sympathetic nerve terminals can also impact renal vasculature and function [26]. Understanding these complex neurological networks could pave the way for targeted therapies for FSGS and other kidney diseases.

Researchers recently discovered a new neural pathway connecting kidney afferent nerves to specific brain regions, including the subfornical organ (SFO) and paraventricular nucleus (PVN) [31]. This pathway was found to be hyperactive in chronic kidney disease (CKD) and heart failure (HF), leading to increased sympathetic activity and worsening the progression of both diseases. Disruption of this circuit has been shown to reduce sympathetic activity and mitigate organ damage, suggesting potential therapeutic targets for CKD and HF [31]. Further research is needed to explore these mechanisms and develop targeted therapeutic approaches for kidney diseases.

The validation of data in this study through pathway analysis of differentially expressed genes in various populations has uncovered an intriguing aspect of FSGS. It was found that distinct cellular pathways are activated in each population. This indicates the complexity of FSGS as a disease, with European patients activating pathways related to muscle contraction and heart processes. In contrast, UAE patients exhibit activation of pathways such as amide biosynthetic process and regulation of cellular stress. Additionally, the UAE population displayed a higher prevalence of *TUBB6*, *RPL27*, and *PFDN5* than the European population. The observed variations in activated pathways and genetic expressions can be ascribed to the genetic diversity across different populations. As documented in the literature, the European population had an upregulation of the following critical genes associated with FSGS: *COL4A5*, *COL4A3*, *NPHS2*, and *TRPC6* [32]. The population of the Arabian Peninsula has demonstrated distinct genetic mutations, which may be due to specific population differences. Notably, within this population, genes associated with nephrotic syndromes include *MYOE1*, *NPHS1*, *NPHS2*, and *PLCE* [33].

The analysis revealed that specific genes (as seen in Figure 5) are shared between the two populations (European and Arabian Peninsula), with *TUBB6* displaying more significant variability in expression levels between healthy individuals and patients. Despite this, both groups are associated with presynaptic pathways, and the shared genes are mainly found in C7 gene sets related to immunology. These findings indicate that FSGS is linked to abnormalities in neural pathways associated with the kidney, particularly the presynaptic pathway that regulates the release of molecules responsible for activating crucial molecular mechanisms in the kidney’s filtration process [34].

Our investigation revealed a higher incidence of FSGS in males, consistent with previous studies [35]. The age range of FSGS patients was 17–65, suggesting that the disease typically manifests in adulthood (Table 5, Table 6 and Table 7). This underscores the significance of considering age and genetic background in diagnosing and treating FSGS. We also noted a significant correlation between FSGS and the Arab population, likely stemming from genetic factors and high consanguinity rates. This highlights the importance of genetic screening and early intervention within these communities. Treatment approaches varied depending on disease severity (Table 6). Severe FSGS was managed with corticosteroids such as prednisone as first-line therapy due to its immunosuppressive and anti-inflammatory effects, and other immunosuppressive medication such as azathioprine [36,37]. Mild cases were treated with SGLT2-inhibitors such as JARDIANCE (empagliflozin) to reduce proteinuria, and ACE-inhibitors such as COVERSYL (perindopril) were administered to lower blood pressure and slow the progression of kidney disease [37,38]. Individualized treatment plans and close monitoring are essential for managing FSGS, especially in populations with varied genetic backgrounds.

The WES detected a novel mutation in the *NFASC* (c.-184T>C) gene. Neurofascin, a member of the L1 family of immunoglobulin cell adhesion molecules, is known for its crucial roles in axon guidance, synaptic formation, and developing the axon initial segment and nodes of Ranvier in the nervous system. Recent research has broadened our knowledge of neurofascin’s functions, revealing its presence in podocytes, specialized cells essential for glomerular filtration in the kidney. Co-localization of neurofascin with podocyte markers in glomerular crescents indicates its potential involvement in podocyte injury and the pathogenesis of kidney diseases [39].

In addition, this research has discovered a previously unreported missense mutation in the *UBXN2B* gene (c.233G>A). *UBXN2B*, UBX Domain Protein 2B, encodes a protein predicted to have ubiquitin-binding capabilities and that plays a role in establishing the orientation of the mitotic spindle. It negatively regulates protein localization to the centrosome and positively regulates mitotic centrosome separation [40]. This protein is primarily localized in the Golgi apparatus, endoplasmic reticulum, and spindle pole centrosome and is also active in the cytosol and nucleus [41].

Another novel missense mutation was found in the *NUDT14* gene (c.146A>T). *NUDT14*, a unique Nudix hydrolase enzyme, is activated by AMPK (AMP-activated protein kinase), an essential cellular energy sensor. This phosphorylation process is anticipated to modulate Nudt14’s interactions with other proteins or its ability to form dimers. Nudt14 is responsible for the specific breakdown of UDP-glucose, which serves as a precursor for glucosylceramide (GlcCer) [42].

Moreover, there is an intronic mutation in *LAMB1* (c.38-28C>A). The gene *LAMB1* encodes the laminin β1 (Lamβ1) protein, which plays a critical role in the kidneys’ GBM structure. The GBM serves as a selective filter, preventing the passage of large molecules, such as proteins, into the urine [43]. Increased expression of *LAMB1* is observed in response to kidney inflammation and serves as a biomarker for diabetic nephropathy, indicating its relevance to kidney injury and repair [44]. This highlights the potential for *LAMB1* as a therapeutic target for certain kidney diseases [43].

The GSEA analysis identified notable genetic expression changes in the *NFASC*, *UBXN2B*, *NUDT14*, and *LAMB1* genes among patients diagnosed with FSGS and MCD. Furthermore, Table 5 outlines additional mutations in various genes that may have associations with FSGS.

In this study, RNA-Seq analysis revealed overexpression of the *TUBB6*, *RPL27*, and *PFDN5* genes. Furthermore, the WES of the FSGS family revealed multiple mutations in these genes present in all affected family members. Interestingly, these mutations were also found in the healthy father, who showed no disease symptoms (Supplementary Figure S1). This observation may be attributed to incomplete penetrance, where some carriers remain unaffected [9]. Therefore, transcriptomics is more effective than genomics in identifying the presence of FSGS. This means that even if patients have the mutation, the disease may only be evident through transcriptome analysis. Additionally, WES detected three genetic mutations in the *NFASC*, *UBXN2B*, *NUDT14*, and *LAMB1* genes, as shown in Figure 9.

In our research, we identified significant genes associated with the development and progression of FSGS. These genes are implicated in the presynaptic pathway present in FSGS and MCD patients, suggesting their potential involvement in kidney and neural pathways. Further investigations are still needed to validate the potential of these genes as biomarkers for detecting FSGS and MCD and to clarify their role in disease development and their association with these conditions.

### Limitations and Future Works

The current study sheds light on the genetic landscape of FSGS but is limited by the small sample size and challenges in obtaining kidney biopsies, which may impact the generalizability of the findings. Although integrating data from related kidney diseases like MCD helped address some limitations, the analysis was confined to kidney tissue and saliva samples. To avoid potential confounding factors, we focused on different affected kidney tissues from patients with FSGS and MCD. We utilized tubulointerstitial tissue from MCD controls with FSGS glomeruli and integrated tubulointerstitial data from FSGS patients to enhance our analysis. It is important for future studies to conduct immunostaining on kidney tissues and include a wider range of tissue types, such as blood and urine, in order to validate the correlation of the identified biomarkers with disease progression.

Moreover, focusing on a specific region and population may limit the applicability of the results to other ethnicities and geographical locations. The functional roles of the potential biomarkers identified have yet to undergo comprehensive validation. We performed RNA sequencing validation experiments in triplicate to validate significant changes, enhance the reliability of our data, and address the constraints of our sample size. Future research should include more extensive, diverse, and international cohorts to address these issues. Functional validation of genetic markers in clinical settings is essential to determine their viability as biomarkers or therapeutic targets. Incorporating functional assays to confirm the biological and clinical relevance of the identified genes will strengthen their potential application in the diagnosis and treatment of FSGS.

## 4. Materials and Methods

### 4.1. Patient Samples

The in silico study involved identifying glomerular (n = 10) and tubulointerstitial tissue (n = 3) from FSGS samples in conjunction with MCD samples (n = 5), shown in Supplementary Table S4. For whole exome sequencing (WES) analysis, five FSGS patients and one non-FSGS control from the same family were recruited for DNA extraction from saliva, see Figure 10. All participants provided informed consent and underwent detailed medical history, physical examination, and laboratory tests to confirm diagnosis and obtain relevant clinical information, see Table 5. The analysis for this study was approved by the Research Ethics Committee of Sharjah University; the ethical approval numbers of the study are REC-15-11-P004 and REC-23-12-08-PG.

**Figure 11:**
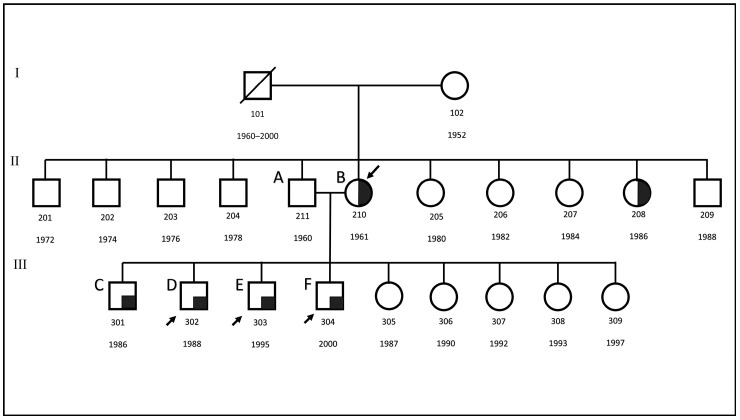
Three-generation pedigree of a UAE Family with FSGS. The proband (↙) is the mother (210) of four affected sons (301–304) with FSGS and five healthy daughters (305–309). Three FSGS patients (↗) from this family were included in the RNA-seq analysis. Participants are labeled A–F, with black representing those with FSGS and white for healthy individuals.

Three FSGS patients from the same family as in Figure 11 and three healthy controls were included for RNA-Seq analysis (Table 5). The samples were collected at the University of Sharjah as part of a research study on the UAE population. After signing the consent form, all participants submitted their medical reports and laboratory results confirming the presence of the disease.

For validation purposes, the study included well-characterized FFPE biopsies of kidney tissues from six FSGS (FSGS 1–6) and seven MCD patients (MCD 1–7) from the UAE population, see Table 7. Further validation was performed using 3 FSGS saliva samples (FSGS7–9) and 7 healthy controls (Control 1–7), as shown in Table 7.

### 4.2. Identifying Activated Cellular Pathways in FSGS Glomerular and Tubulointerstitial Tissue

The gene expression list of glomerular and tubulointerstitial tissue was analyzed using absolute GSEA [45] to identify the activated and enriched cellular pathways. This was carried out by performing absolute GSEA on the expression data using around 33,493 annotated cellular pathways obtained across seven well-annotated gene sets (C1, 2, 3, 5, 6, 7, and 8) found in the Broad Institute’s database (https://www.gsea-msigdb.org). The pathways that showed significant activation in both sample types were identified using a threshold of *p* < 0.05 and FDR < 0.25 [46,47]. This criterion was applied to ensure reliable and consistent results in the analysis. The enriched genes between glomerular and tubulointerstitial tissue were identified by processing the selected pathways. The set of available genes was reduced by checking the frequency of gene occurrence across differentially activated cellular pathways. This method helped to identify the explicitly activated cellular pathways in FSGS patients compared to MCD patients.

### 4.3. Analyses of Publicly Available Transcriptomic Data Sets for FSGS

To elucidate the biological cellular pathways and differentially expressed genes associated with FSGS, transcriptomic data sets of FSGS were searched and retrieved from gene expression omnibus (GEO). The obtained data sets were exclusively from the Affymetrix Human Genome Plus 2.0 Array (GPL570) platform to minimize potential platform-induced bias. Subsequently, one data set, GSE99340, met these criteria encompassing FSGS relevance and origin from the GPL570 platform. The raw data and probe annotation files were then procured for comprehensive transcriptomic analysis.

### 4.4. Data Analysis Using Affymetrix Microarray and R Software Packages

The study utilized Affymetrix microarray, which had a total of 54,675 probes. Raw CEL files were obtained from the GEO database and processed using an in-house R script. The data were normalized, and background noise was removed, as described earlier [46]. Analysis was performed using R software packages (4.3.2 and 2.8.0). Gene Chip Robust Multiarray Averaging (GCRMA) and Affymetrix Microarray Suite 5 (MAS5) are two standard methods used in gene expression analysis. These methods process and normalize raw microarray data for accurate and reliable gene expression measurements. Invariant probes were removed, and non-specific filtering was performed to obtain a standard set of variant probes. An adaptive filtering technique was used to eliminate probes with a MAS5 value greater than 50 and a coefficient of variation (CV) between 10 and 100% in GCRMA across all cases.

The resulting filtered probes were mapped to the gene list using the Broad Institute software “http://software.broadinstitute.org/gsea/downloads.jsp (accessed on 24 January 2024)” [45]. The analysis excluded probes that corresponded to housekeeping genes or were unassigned to any gene. The expression value for each gene was determined by selecting probes with the highest expression level.

### 4.5. Differential Gene Expression in Glomerular Tissue Compared to Tubulointerstitial Tissue

Two different methods were used to perform the differential gene expression analysis. These methods provided information based on pathway enrichment and microarray gene expression. As described earlier [46], the R script was employed to obtain genes that occurred frequently across all enriched pathways for each sample set. The 95th percentile cut-off was calculated for each sample using statistical analysis.

### 4.6. RNA Sequencing of FSGS and Healthy Control Samples

RNAs were extracted from the saliva samples using the RNeasy kit (Qiagen, Hilden, Germany) according to the manufacturer’s instructions. Genomic DNA removal from all RNA extractions was carried out by treating the extracted RNA with a TURBO DNAase-free TM Kit (Invitrogen, Carlsbad, CA, USA). The extracted RNAs were used for whole transcriptome sequencing using AmpliSeq Whole Transcriptome on the S5 System (Thermo (Fisher) Scientific, MA, USA), as previously described [48]. The targeted RNA-seq library was prepared using the Ion AmpliSeq Transcriptome Human Gene Expression Kit (Thermo Fisher Scientific), which could profile over 21,000 distinct human RNA targets. The resulting template libraries were then sequenced on the Ion S5 XL Semiconductor sequencer using the Ion 540 Chip (Life Technologies Corporation, Carlsbad, CA, USA) prepared on the fully automated Ion Chef System (Thermo Fisher Scientific).

### 4.7. RNA-Seq Data Analysis

The RNASeq data was analyzed using Ion Torrent Software Suite version 5.4. Alignment was performed using the Torrent Mapping Alignment Program (TMAP) optimized for Ion Torrent sequencing data, aligning raw sequencing reads against the reference sequence derived from the hg19 (GRCh37) assembly. Differential gene expression (DGE) analysis was conducted using the R/Bioconductor package DESeq2 with raw read counts from RNA-seq data. Genes with less than ten normalized read counts were excluded from further analysis. Differentially expressed genes were identified at a significance level of *p* < 0.05.

### 4.8. Gene Expression Assessment via Quantitative Reverse Transcriptase Polymerase Chain Reaction (qRT-PCR)

Three sequential sections, at 3 µm, from formalin-fixed paraffin-embedded (FFPE) tissue biopsies from the cases mentioned in Table 7 were subjected to RNA extraction using the Recover All kit according to the manufacturer’s instructions (Thermo (Fisher) Scientific, MA, USA). The highly expressed 4 candidate biomarkers, according to the in silico and RNA sequencing results, were chosen for further quantitative gene expression analysis comparing FSGS and MCD patients from the UAE population. Complementary DNA (cDNA) was synthesized using a High-Capacity cDNA Reverse Transcription Kit (Applied Biosystems, Waltham, MA, USA) following the manufacturer’s instructions.

Gene expression was determined by quantitative PCR (qPCR) using Maxima SYBR Green/ROX qPCR MasterMix (2×) (Thermo Scientific, USA) on a QuantStudio3 Real-Time PCR thermal cycler (Applied Biosystems). qPCR runs were performed using the primer sets as detailed in Supplementary Table S5.

Each sample underwent amplification in two biological and two technical replicates. The mean quantitation cycle (Cq) value was utilized to determine the fold expression change in FSGS patients compared to those with MCD. The relative expression levels of the target gene were calculated using the 2^−ΔΔCt^ method.

### 4.9. Whole Exome Sequencing (WES)

Total DNAs were extracted from saliva samples using the Oragene-DNA Kit (OG-500, DNA Genotek, Stittsville, ON, Canada) according to the manufacturer’s instructions. WES was performed on an Illumina HiSeq 2500 system (Illumina, San Diego, CA, USA) using the extracted genomic DNA. The resulting data were processed using standard procedures provided by Genetics-India. The paired-end reads of 2 × 100 bases, which passed quality control (Phred score of > 20), were aligned to the human reference genome build hg19/GRCh37 using BWA [49]. The resulting BAM and VCF files were processed using SAM tools, and variants were identified using the Genome Analysis Tool Kit (GATK) v2.7.2 [50]. The identified variants were annotated using dbSNP (build 137) and filtered at a read depth of ≥10. Variants that passed this filter were further filtered by their frequency in dbSNP, ExAC Browser, gnomAD, 1000Genomes Project, or Ensembl. We utilized the SureSelect All Exon V5 kit from Agilent Technologies (Agilent Technologies, Santa Clara, CA, USA) to perform exome capture and enrichment. Synonymous and common SNPs were filtered out, and rare variants were considered potential disease-causing candidates, as previously reported [51].

## 5. Conclusions

Analyzing gene pathways in diverse populations revealed shared and unique FSGS pathways. Understanding these gene-mediated pathways can improve prognostic assays and develop molecular inhibitors, opening up new treatment options and diagnostic strategies. Our research has identified previously unreported genes linked to FSGS, namely, *RPL27*, *TUBB6*, and *PFDN5*. To our knowledge, this is probably the first study that has investigated the genetics of FSGS across different populations. This discovery represents a novel finding with potential implications for the early detection of FSGS and may pave the way for further advancements in transitional medicine.

## Figures and Tables

**Table 1:** List of number of significant pathways enriched in glomerular tissue compared to tubulointerstitial tissue in absolute GSEA.

Gene Set Analyze	Description	Total Number of Pathways from MSigdb	Total Number of Significant Pathways from absGSEA
C1	Positional gene sets	301	6
C2	Curated gene sets	7233	53
C3	Regulatory target gene sets	3713	126
C5	Ontology gene sets	16,008	229
C6	Oncogenic signature gene sets	189	20
C7	Immunologic signature gene sets	5219	131
C8	Cell type signature gene sets	830	23

C4 was excluded due to the lack of relevance to the disease. MSigdb: molecular signatures database.

**Table 2:** List of the top 40 genes based on frequency in MCD versus FSGS set.

C5		C7	
Gene	Frequency	Gene	Frequency
KCNJ2	62	GIMAP4	10
ATP1A2	53	LIMK2	10
GJA5	50	ANXA1	9
KCND3	50	HEBP2	8
SCN2B	48	NCAM1	8
CACNA1D	44	RBP4	8
CACNB2	44	RIPOR2	8
KCNH2	44	ABCD3	7
KCNJ3	43	ACVR1B	7
ADORA1	39	ADARB1	7
SNCA	37	ADGRE5	7
DSG2	36	IL18BP	7
ITGB1	35	LDB2	7
PTPRO	35	MRC2	7
NPHS1	31	NXN	7
GRIK2	30	ACADL	6
PDE4B	29	AOPEP	6
DLG1	28	ARRB1	6
TRPC6	28	CAMK1D	6
ADORA2A	27	CDHR5	6

**Table 3:** List of the top 20 genes based on frequency in healthy control versus FSGS set from the UAE population.

C5		C7	
Gene	Frequency	Gene	Frequency
RPL26	7	PFDN5	2
EEF2K	6	RPL6	2
MOV10	6	RAD52	1
FASTKD3	6	MYO9A	1
MTIF2	6	PABPC3	1
MAPKAPK5	6	SNRPD2	1
RPL27	5	MARCKS	1
RPL35	5	EEF2K	1
RPS19	5	MOV10	1
RPL39	4	RPL39	1
TGFB2	3	SLC12A4	1

**Table 4:** WES identifies significant novel mutations among patients with FSGS.

Gene	Variant Class	cDNA Change	Amino Acid Change
NFASC	5UTR	c.-184T>C	NA
DSTYK	MISSENSE	c.2747C>T	p.Ser916Phe
CAV3	MISSENSE	c.233C>T	p.Thr78Met
SLC6A11	SILENT	c.717T>C	p.Cys239=
SYN2	MISSENSE	c.118C>T	p.Pro40Ser
ACHE	SILENT	c.768C>T	p.Ala256=
SRPK2	SILENT	c.1350A>G	p.Pro450=
PIK3CG	MISSENSE	c.1613C>T	p.Pro538Leu
LAMB1	INTRONIC	c.38-28C>A	p.?
UBXN2B	MISSENSE	c.233G>A	p.Arg78Gln
TRIP11	SILENT	c.2397T>C	p.Ser799=
SERPINA12	MISSENSE	c.937A>G	p.Met313Val
SERPINA12	MISSENSE	c.247T>G	p.Phe83Val
SERPINA4	MISSENSE	c.155C>T	p.Ala52Val
HSP90AA1	INFRAME-DEL	c.739_744del	p.Lys247_Glu248del
EXOC3L4	MISSENSE	c.88C>T	p.Arg30Trp
NUDT14	MISSENSE	c.146A>T	p.Asn49Ile
ATP2C2	MISSENSE	c.1123G>A	p.Val375Ile

NA; Not identified, (=); Silent mutation, no amino acid change; (?); Unknown amino acid change.

**Table 5:** Clinical description of all the participants from a UAE family with FSGS, including their disease stage, gender, age, nationality, ethnicity, and symptoms. P-ID; participant identification.

P-ID	Disease Stage	Gender	Age	Nationality	Ethnicity	Symptoms
A	Unaffected	Male	64	Emirati	Arabian Peninsula	Cardiovascular diseases
B	Advanced stage of FSGS	female	63	Emirati	Arabian Peninsula	Renal Failure/Renal transplantation
C	Advanced stage of FSGS	Male	38	Emirati	Arabian Peninsula	Renal Failure/Renal transplantation
D	Advanced stage of FSGS	Male	36	Emirati	Arabian Peninsula	Renal Failure/Kidney dialysis
E	Mild stage of FSGS	Male	29	Emirati	Arabian Peninsula	Proteinuria and Hematuria
F	Mild stage of FSGS	Male	24	Emirati	Arabian Peninsula	Proteinuria and Hematuria

**Table 6:** Clinical description of participants in the RNA sequencing analysis, including their gender, age, nationality, ethnicity, disease stage, and medications.

Sample No	Gender	Age	Nationality	Ethnicity	Disease Stage	Medications
FSGS1	Male	37	UAE	Arabian Peninsula	Advanced stage of FSGS	Corticosteroids/Immunosuppressives
FSGS2	Male	28	UAE	Arabian Peninsula	Mild stage of FSGS	ACE-inhibitor/SGLT2-inhibitor
FSGS3	Male	25	UAE	Arabian Peninsula	Mild stage of FSGS	ACE-inhibitor/SGLT2-inhibitor
Control 1	Male	39	UAE	Arabian Peninsula	Healthy individuals	No medication
Control 2	Male	37	UAE	Arabian Peninsula	Healthy individuals	No medication
Control 3	Male	25	UAE	Arabian Peninsula	Healthy individuals	No medication

ACE-inhibitor; angiotensin-converting enzyme inhibitor; SGLT2-inhibitor; sodium-glucose co-transporter 2 inhibitor.

**Table 7:** Clinical details of participants in the validation using qRT-PCR, including their gender, age, nationality, ethnicity, disease description, and source of tissue sample.

Sample No	Gender	Age	Nationality	Ethnicity	Disease Description	Source
FSGS1	Female	65	UAE	Arabian Peninsula	Diffuse lesions of diabetic glomerulosclerosis	FFPE
FSGS2	Male	NA	UAE	Arabian Peninsula	Severe advanced stage of FSGS	FFPE
FSGS3	Male	17	UAE	Arabian Peninsula	FSGS and associated with mesangial hypercellularity and severe tubulointerstitial reaction.	FFPE
FSGS4	Female	28	UAE	Arabian Peninsula	FSGS	FFPE
FSGS5	Female	24	UAE	Arabian Peninsula	Moderate stage of FSGS	FFPE
FSGS6	NA	NA	UAE	Arabian Peninsula	FSGS	FFPE
FSGS7	Male	37	UAE	Arabian Peninsula	Severe advanced stage of FSGS	Saliva
FSGS8	Male	28	UAE	Arabian Peninsula	Mild stage of FSGS	Saliva
FSGS9	Male	25	UAE	Arabian Peninsula	Mild stage of FSGS	Saliva
MCD1	Male	4	UAE	Arabian Peninsula	MCD with mesangial hypercellularity	FFPE
MCD2	Male	35	INDIA	South Asian	MCD	FFPE
MCD3	Female	24	INDIA	South Asian	MCD with mesangial hypercellularity	FFPE
MCD4	Female	9	Pakistan	South Asian	MCD	FFPE
MCD5	Male	14	IRAQ	Arabian Peninsula	MCD with mesangial hypercellularity (no response to steroid therapy).	FFPE
MCD6	Male	23	Bangladesh	South Asian	MCD with mesangial hypercellularity.	FFPE
MCD7	Female	34	INDIA	South Asian	Minimal change disease with mesangial hypercellularity.	FFPE
Control 1	Male	39	UAE	Arabian Peninsula	Healthy individual	Saliva
Control 2	Male	37	UAE	Arabian Peninsula	Healthy individual	Saliva
Control 3	Male	25	UAE	Arabian Peninsula	Healthy individual	Saliva
Control 4	Male	42	UAE	Arabian Peninsula	Healthy individual	Saliva
Control 5	Male	8	UAE	Arabian Peninsula	Healthy individual	Saliva
Control 6	Female	10	UAE	Arabian Peninsula	Healthy individual	Saliva
Control 7	Female	31	UAE	Arabian Peninsula	Healthy individual	Saliva

## Data Availability

The raw RNA sequencing data generated in this work can be accessed from supplementary methods. The data from publicly available sources used for in silico analysis in this study can be accessed from Gene Expression Omnibus (GEO) under GEO Series access number GSE99340 and can be accessed from https://www.ncbi.nlm.nih.gov/geo/query/acc.cgi?acc=GSE99340 (accessed on 25 January 2024)”. All other supporting data of this study are either included in the manuscript or available on request from the corresponding author.

## Supplementary Materials

The following supporting information can be downloaded at: https://www.mdpi.com/article/10.3390/ijms252111659/s1.
