# Supplementary figures and images for: Identification of Genes Associated with Familial Focal Segmental Glomerulosclerosis Through Transcriptomics and In Silico Analysis, Including RPL27, TUBB6, and PFDN5
Source: Int J Mol Sci. 2024 Oct 30;25(21):11659. doi: 10.3390/ijms252111659 (PMC11546068; doi:10.3390/ijms252111659)

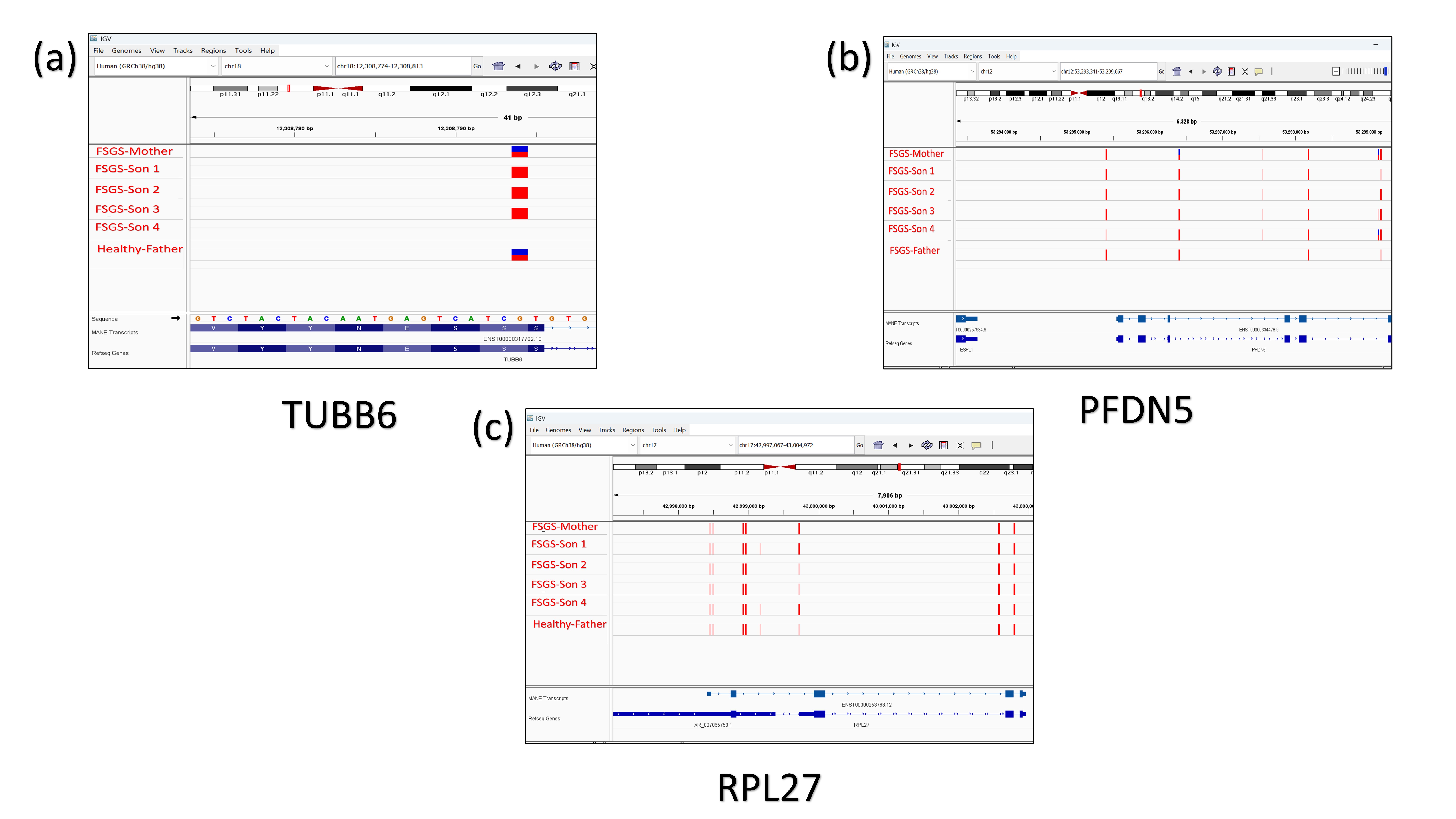

Supplement: Supplementary file 1 [file ijms-25-11659-s001.zip › Figure S1.jpg]
